# Preparation of Layer-by-Layer Nanofiltration Membranes by Dynamic Deposition and Crosslinking

**DOI:** 10.3390/membranes9020020

**Published:** 2019-01-24

**Authors:** Yan Liu, George Q. Chen, Xiuli Yang, Huining Deng

**Affiliations:** 1 School of Chemical Engineering and Technology, Hebei University of Technology, Tianjin 300130, China; julia_liuyan@hebut.edu.cn (Y.L.); xiuliyang99@sina.com (X.Y.); 2 Department of Chemical Engineering, The University of Melbourne, Parkville, VIC 3010, Australia; gechen@unimelb.edu.au

**Keywords:** nanofiltration membrane, polyelectrolyte, dynamic deposition, crosslinking

## Abstract

In recent decades, the advancements in layer-by-layer (LBL) assembly technology have provoked increasing interest in the preparation of multilayer polyelectrolyte membranes with excellent performance. In the current study, a novel nanofiltration (NF) membrane was prepared by pressure-driven layer-by-layer (LBL) assembly of polyethylenimine (PEI) and polyacrylicacid (PAA) on a porous substrate with chemical crosslinking. The effect of deposition pressure on separation performance of the prepared membranes was studied. The surface morphology, hydrophilicity and the charge property of the dynamically-deposited membranes were compared with those prepared by static adsorption. The characterization results showed that dynamic deposition process resulted in a smoother membrane surface with improved hydrophilicity. The mechanism of water-path formation was proposed to interpret the effect of pressure on the membrane performance. Glutaraldehyde (GA) was used as a crosslinker to reduce the number of polyelectrolyte bilayers for obtaining good separation performance. The rejections of different inorganic salts of the dynamically-deposited NF membrane were also investigated.

## 1. Introduction

As a pressure-driven membrane process, nanofiltration (NF) has been applied in a wide range of fields, such as water softening, brackish water reclamation and large molecular removal [1,2,3,4,5,6]. Most of these applications require membranes with both high permeability of water and high retention of solutes (i.e., high productivity with good selectivity). However, a trade-off effect between the enhancement of the solute rejection and water flux of membranes has been observed for many types of NF membranes [7,8,9]. For example, selectivity can be improved by increasing the thickness of the skin layer, accompanied by a decreased water flux.

The preparation methods of NF membrane mainly include phase inversion [10,11], interfacial polymerization [12], grafting [13] and film deposition on a preformed substrate [14]. Most of the methods prepare membranes with negatively charged surface. The preparation of positively charged NF membranes would further extend their applications, such as multivalent cation removal. Layer-by-layer (LBL) assembly, which was initially proposed by Decher in 1997 [15], could be used to regulate surface charge by changing the terminated polyelectrolyte. In the past two decades, the multilayer assembly technology has witnessed considerable growth owing to its simple operation and thickness-tuning capacity at the nanoscale [16]. Most studies have investigated LBL self-assembly by adsorption, which is performed by membrane immersion and rinsing in the selected electrolyte solutions [17,18,19,20]. Several recent reports showed that dynamic LBL self-assembly could effectively reduce the number of deposition cycles and obtain membranes with improved performance [21,22,23]. The dynamic assembly process is more efficient than the traditional adsorption method, as fabricated membranes with less than five bilayers showed even better selectivity than static membranes with dozens of bilayers [22]. However, most of studies focused on the high efficiency of the dynamic process. Dynamic assembly NF membranes were deposited at a fixed pressure [21], and a few works have focused on the effects of pressure applied during layer deposition on the separation performance of NF membranes.

Chemical crosslinking has been adopted by researchers to tune the structure of multilayer polyelectrolytes, and thus improve the tightness of NF membranes [24,25,26]. Wang et al. [24] cross-linked with a LBL polyelectrolyte layer with glutaraldehyde (GA) to develop NF membranes for water softening, and found that the retention of the prepared membranes was mainly based on the size exclusion effect. Crosslinking has also been proven to decrease the swelling effect of polyelectrolyte, enhancing solvent and pH resistances [27,28].

The current study aims to develop novel positively-charged NF membranes with dynamic deposition and chemical crosslinking approaches that exhibit improved separation performance with only 1.5 bilayers of deposited polyelectrolytes. This study assembles polyelectrolyte multilayer membranes with different deposition pressures. The enhancement in the performance of dynamic membranes is compared against the statically-adsorbed ones. The characteristics and mechanisms of performance improvements of the dynamically-assembled membranes are discussed.

## 2. Experimental Section

### 2.1. Materials

Sepro Company supplied the polyacrylonitrile (PAN) substrates with a molecular weight cutoff (MWCO) of 50,000. Sodium hydroxide (NaOH, Tianjin DaMao, Tianjin, China) was used as the alkaline solution for the hydrolysis of the PAN substrates to enhance the surface charge density and hydrophilicity. Polyethylenimine (PEI, 50 wt%, *Mw* = 70,000, Aladdin) and polyacrylicacid (PAA, 25 wt%, *Mw* = 240,000, Acros) solutions were used as polyelectrolytes for the formation of modified layers on the PAN substrates. Glutaraldehyde (GA), epichlorohydrin (ECH) and ethanol, provided by Tianjin Guangfu Fine Chemical Research Institute, were used as cross-linkers. Multilayer membrane performance was determined by magnesium chloride (MgCl_2_), magnesium sulfate (MgSO_4_) and sodium chloride (NaCl), which were obtained from Tianjin DaMao Chemical Reagent Company (analytical grade).

### 2.2. Preparation of Membranes

#### 2.2.1. Support Membrane Modification

The PAN substrates were immersed in a 2 M NaOH solution at 65 °C for 30 min to convert the nitrile (–C≡N) groups of the PAN to carboxylate(–COOH). The membranes were taken out and soaked in distilled water until the pH values of the distilled water reached about 7.0. This hydrolysis process resulted in high negative charge density for the PAN support membranes.

#### 2.2.2. Multilayer Polyelectrolyte Membrane Preparation by Static Adsorption

The pretreated PAN substrates (8 × 8 cm^2^) were alternately immersed into 3 g/L PEI and 0.6 g/L PAA for 30 min. After each deposition step, the membranes were taken out and rinsed with distilled water. Thus, one bilayer of self-assembly membrane was completed. The adsorption procedure was repeated until the desired bilayer number was reached.

#### 2.2.3. Multilayer Polyelectrolyte Membrane Preparation by Dynamic Deposition

An apparatus made in house carried out the preparation of polyelectrolyte multilayer membrane under pressure. The support membrane was fixed into a cell and a nitrogen cylinder provided the pressure. The process of pressure deposition LBL included the following preparation procedure: (a) 3 g/L PEI (35 mL) was poured into the membrane cell and was pressurized through the membrane under 0.2 MPa for 30 min, (b) the membrane was rinsed and pressurized under 0.2 MPa for 10 min using distilled water, (c) 0.6 g/L PAA (35 mL) was deposited and rinsed in a similar way as step (b). These steps prepared additional bilayers until the target number of bilayers was produced.

In the process of dynamic deposition, the initial PEI concentration before deposition, the feed and permeate after deposition were determined by a UV-Vis spectrophotometer with copper nitrate as the chromogenic agent. PEI deposition amount Q was calculated using the following equation:

$$Q=\frac{C_{0}V_{0}-\left(C_{1}V_{1}+C_{2}V_{2}\right)}{A}$$

where *V*_0_, *V*_1_ and *V*_2_ are the volume of the initial solution before deposition and the feed and permeate after deposition, respectively. *C*_0_, *C*_1_ and *C*_2_ are the PEI concentration of the initial solution before deposition and the feed and permeate after deposition, respectively. *A* is the effective deposition area of the membrane.

In this study, 0.5-bilayers refer to the membrane as only having a layer of PEI deposited and 1.5-bilayers represent the membrane had one PEI/PAA bilayer and terminated with another layer of PEI. The PAA-terminated membrane had an integral number of bilayers.

#### 2.2.4. Cross-Linking

The modified membrane was firstly heat-treated at 80 °C to dry and preliminarily stabilize the polyelectrolyte layers. It was then chemically cross-linked by immersing the membranes into a 3 wt% GA aqueous solution for 20 min.

### 2.3. Surface Characterization

Scanning electron microscopy (SEM, FEI, Nova Nano SEM450, Hillsboro, OR, USA) was used to characterize the surface morphology of the prepared multilayer membranes.

The water contact angle of the composite membranes was performed at room temperature to evaluate the surface hydrophilicity by contact angle measurements (Krüss, DSA100, Karlsruhe, Germany). The photographs of the contact angle were taken less than 5 s after the water droplet was dropped onto the surface of the modified membranes. Three measurements were taken at different locations of the samples.

Membrane surface zeta potential was acquired by using an electrokinetic analyzer (SurPASS, Anton Paar, Graz, Austria) and 1 mmol/L KCl was selected as the electrolyte solution.

### 2.4. Membrane Performance Test

**Figure 1:**
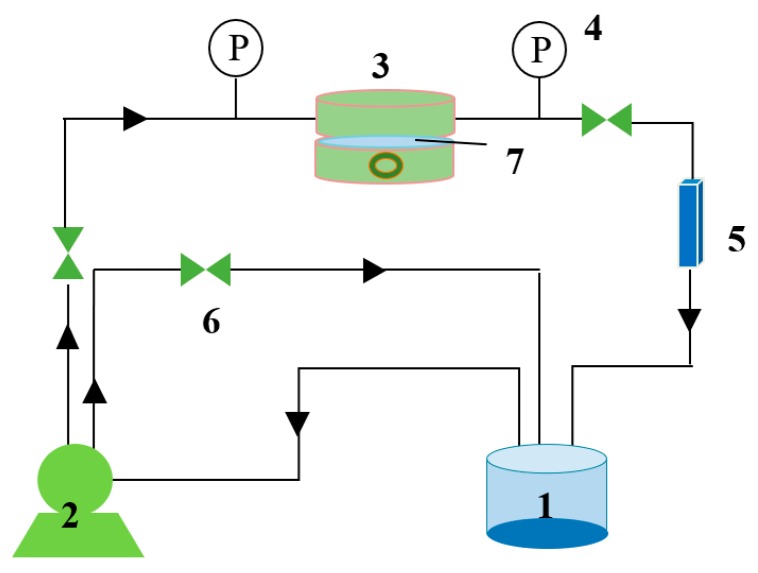
A flowchart of the membrane performance evaluation apparatus. (1. feed tank, 2. pump, 3. membrane module, 4. pressure gauges, 5. flow meter, 6. valve, 7. membrane).

The separation performance of the prepared membrane was performed using a homemade cross-flow apparatus, as shown in Figure 1. The membrane cell has an effective surface area of 38.47 cm^2^. The feed solution was pressurized by a diaphragm pump to 0.4 MPa and circulated back to the feed tank. The volumetric flow rate of the feed solution was 40 L/h. MgCl_2_, NaCl and MgSO_4_ solutions at 0.01 M were selected to evaluate the membrane separation performance. All experiments were performed after a stabilization time of 1 h at 0.4 MPa.

The permeation flux (*J*) was calculated as:

$$J=\frac{\Delta V}{A\times \Delta t}$$

where ΔV is the volume of the permeate during the time interval Δt, and A is the effective membrane area. The salt rejection (*R*) was evaluated as

$$R=\frac{C_{F}-C_{P}}{C_{F}}\times 100\%$$

where $C_{F}$ and $C_{P}$ are the solute concentrations in the feed and the permeate, respectively.

## 3. Results and Discussion

### 3.1. Characteristics of the Multilayer Membranes

**Figure 2:**
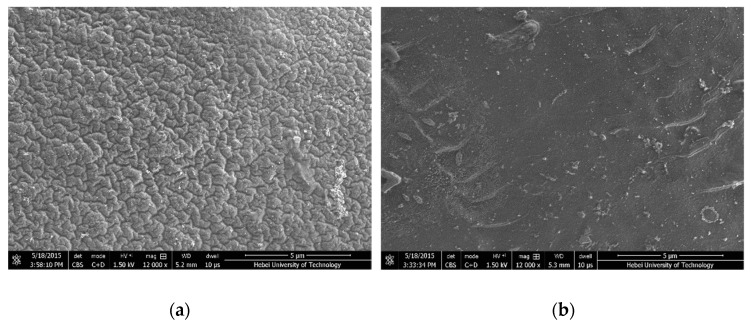
Scanning electron microscope (SEM) images of the multilayer membranes prepared by (**a**) static adsorption, and (**b**) dynamic deposition, respectively, with 1.5-bilayers of polyelectrolytes.

The 1.5-bilayer membranes prepared by static adsorption and dynamic depositions were both cross-linked with GA to achieve a stable structure. Figure 2 shows that, at the same magnification, the surface morphologies of membranes deposited with static and dynamic procedures were different. The surface of the membrane prepared by static adsorption was rough and uneven, while the dynamically-assembled membrane showed a relatively uniform and smooth surface. These results indicate that the dynamic deposition process smoothed the multilayer surface, and a more homogeneous structure might be formed under pressure.

**Figure 3:**
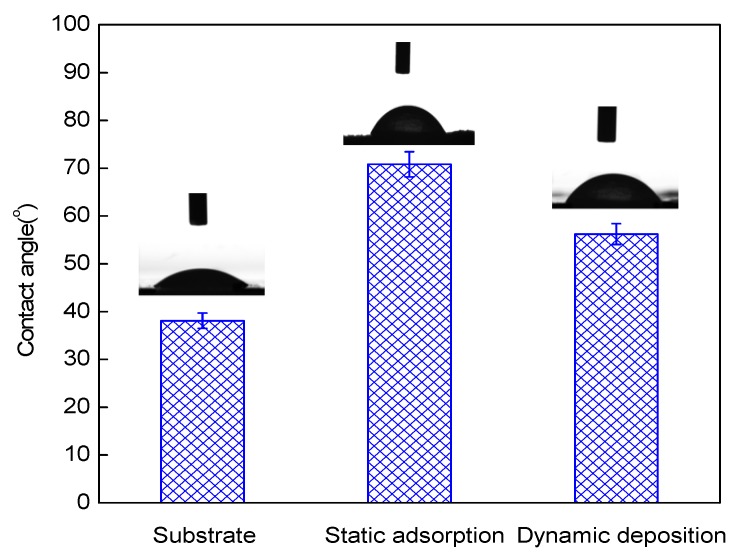
Contact angles of the substrate membrane and membranes prepared with static adsorption and dynamic deposition with 1.5 bilayers of polyelectrolyte.

**Figure 4:**
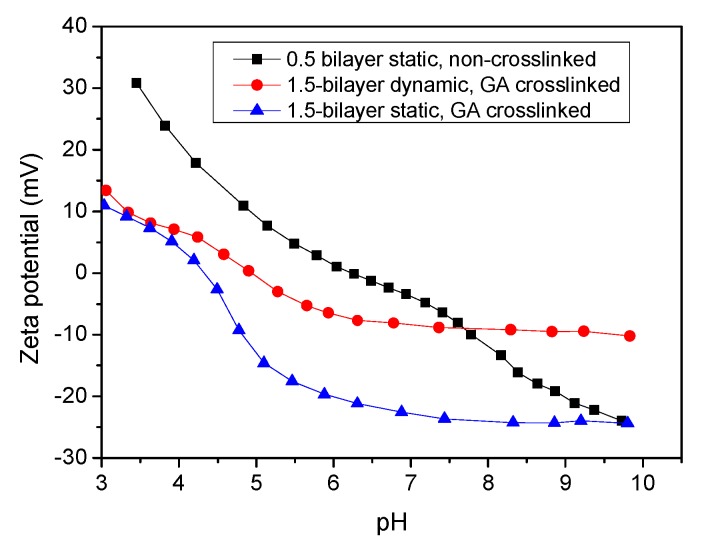
The zeta potential of multilayer membranes.

Water contact angles were used to evaluate the hydrophilicity of the membranes [29]. As seen in Figure 3, the hydrolyzed PAN substrate membranes showed good hydrophilicity with a contact angle of 38.1° due to the existence of carboxyl groups. The contact angle of the 1.5-bilayer membrane increased, which was due to the more hydrophobic property of the terminal PEI on the surface of the membrane. Further, the membranes prepared with dynamic deposition exhibited a lower contact angle (56.2°) compared to those prepared by static adsorption (70.8°). This could partly be attributed to the change of surface morphology of the membranes [30], as shown in Figure 4. Previous studies already proved that the hydrophilicity of membranes plays an important role in water transport resistance in NF separation [31]. The dynamically-assembled membranes are expected to provide improved flux and antifouling performance [32].

The zeta potential results of the 1.5-bilayer cross-linked membrane, prepared with static and dynamic processes are presented in Figure 4. The charge property of the multilayer membranes exhibited typical ampholytic behaviors [24,33]. The surfaces of both membranes were positively charged at a low pH, due to the ionization of amine groups of PEI molecules, but at high pH values, the PEI on the surface turned to almost no-charge and the zeta potentials of membranes were a little less than zero. This should be caused by the dissociation of some carboxyl group of PAA from the inter-diffusion of polyelectrolyte chains [24,33]. Cross-linking with GA also reduced the zeta potential of membranes to some degree because of the transformation of amine to weaker basic groups [24,33]. The dynamically-prepared membrane possessed higher zeta potential than the static one, indicating that the deposition driven by pressure produced a thicker polyelectrolyte layer and improved the efficiency of multilayer formation.

The change of zeta potential of the dynamic membrane with PEI terminated without crosslinking upon the variation of pH and is also shown in Figure 4. During the dynamic assembly process, PEI molecules aggregated on the surface of the substrate membrane and introduced an obvious higher zeta potential than the 1.5-bilayers cross-linked membranes at low pH values. However, it exhibited a severe decrease at alkaline pH values. The main interaction force between the positively charged PEI molecules and the negatively charged substrate surface is electrostatic attraction. As a weak polyelectrolyte, PEI exhibits a high positive charge density at a low pH value, while under alkaline conditions, and the amine groups of PEI tend not to dissociate. Thus, the PEI layer was loosely positioned on the surface of the membrane and was easily washed away during the zeta potential test with a high solution flow rate. As a result, the negative charge resulted from the ionization of carboxyl group and the substrate membrane was monitored gradually. This disassembly of polyelectrolyte membranes with strong acid or basic solution has been reported to be a novel approach for regeneration of fouled membranes [34,35,36]. As the cross-linked membranes exhibit a stable zeta potential at a pH higher than 7, crosslinking is necessary for constructing stable multilayer NF membranes.

### 3.2. Effect of Cross-Linking on Separation Performance

**Figure 5:**
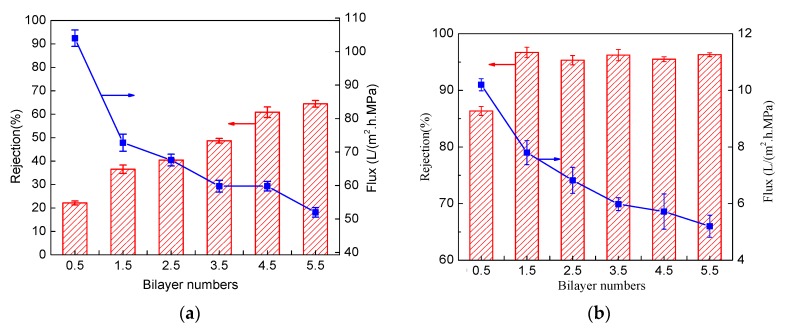
The separation performance of the statically prepared membranes, (**a**) non-cross-linked, and, (**b**) cross-linked with 3 wt% glutaraldehyde (GA) aqueous solution. The crosslinking was conducted for 20 min.

The statically-prepared membranes with different bilayers, with and without GA cross-linking, were prepared, and the performance is depicted in Figure 5. For multilayer membranes without crosslinking, the rejection of MgCl_2_ increased with the deposition bilayers because the more deposited number of polyelectrolyte layer produced a thicker active layer, and thus improved the membrane selectivity. For the same reason, the flux showed a slow decline with the deposited number of layers. The non-cross-linked multilayer membranes showed the highest rejection of MgCl_2_ (less than 70%) even with 5.5 bilayers. Consequently, depositing of more bilayers is needed to get a NF membrane that can be used for practical applications.

For the cross-linked static deposited multilayer membranes, the MgCl_2_ rejection had an obvious improvement. Even with only one layer of PEI deposited, the rejection reached 86%, which is nearly four times that without crosslinking. With a bilayer number greater than 1.5, the membrane exhibited an MgCl_2_ rejection of about 95% (Figure 5). This rejection value is higher than the results reported by Jin et al. with 60 bilayers of polyvinylamine (PVA) and polyvinylsulfate (PVS) on porous materials [37]. This illustrates that cross-linking could increase the salt rejection of a multilayer membrane and reduce the bilayer number to obtain membranes with desired retention. With crosslinking, the enhancement effect of rejection with the increase of the bilayer number was not as obvious. Moreover, the flux exhibited an obvious decline compared to the originals, and a further decrease with the deposited bilayer number. Since the cross-linked membranes with 1.5 bilayers were observed to reach a salt rejection of about 95%, 1.5 bilayers could be deemed as sufficient for fabricating a NF membrane for potential water treatment applications.

### 3.3. Effect of Pressure on Separation Performance of Membranes

**Figure 6:**
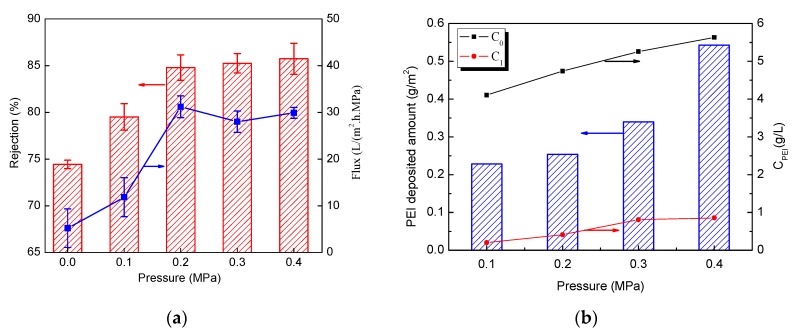
The effect of deposition pressure on (**a**) the performance of the 1.5-bilayers membranes cross-linked with GA and (**b**) the deposited polyethylenimine (PEI) amount. (*C*_0_: retentate concentration, *C*_1_: permeate concentration).

The effect of deposition pressure on the performance of the 1.5-bilayer membranes was investigated. After the dynamic deposition, all the tested membranes were cross-linked with 3 wt% GA for 20 min. As shown in Figure 6a, the rejection of the membranes to MgCl_2_ shows an increase with the depositing pressure at low pressure, but with the pressure higher than 0.2 MPa, the rejection reaches a plateau. Meanwhile, the flux of membranes also rises at low pressures, exhibiting similar trends with the rejection. This is not in accordance with the static adsorption prepared membranes, which typically show a trade-off between flux and rejection [24]. The amount of PEI deposition on the substrate membrane was determined and it was found that more polyelectrolyte was deposited on the membrane surface with increasing pressures (Figure 6b). The increased deposition polyelectrolyte amount explains why an improvement in retention of the dynamically-assembled multilayer membranes was observed.

**Figure 7:**
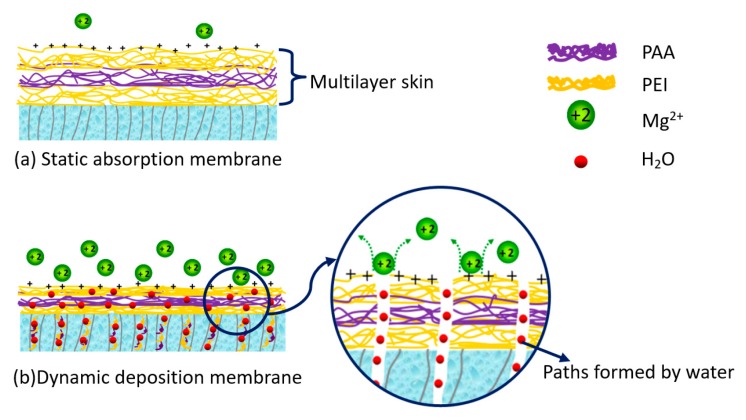
The schematic of static adsorption and dynamic deposition mechanism.

Based on the discussions above regarding membrane characterization and crosslinking, a possible mechanism of dynamic assembly process is proposed in this work to understand the flux enhancement with a higher amount of PEI deposition. In a static adsorption procedure, the self-assembly multilayer skin is formed on the surface of the support membrane with the thickness increasing with the number of deposition layer (Figure 7a). Hence, a thicker or denser skin structure is produced, resulting in improved salt rejection, but reduced water flux. During the dynamic procedure, more polyelectrolytes were deposited with the increase of pressure, as shown in Figure 6b, and higher pressure compacted the multilayer to a denser structure. These could explain the improvement of salt rejection with the increase of deposition pressure. Meanwhile, water in the polyelectrolyte solution could transport through the membrane as driven by pressure, which can form special paths for water transfer through the relatively soft polyelectrolyte layer (Figure 7b). More of these pathways could be constructed as pressure increased, so water permeation is improved. When the deposition pressure further increases to more than 0.2 MPa, the increased amount of polyelectrolyte deposition and the compression effect would obstruct the transport of water and lead to a decline in flux. Therefore, there is a balance between the amount of polyelectrolyte deposition and water paths formed with the depositing pressure.

To further verify this water-path-forming mechanism, the pressure was also used in static deposited membrane preparation. Pressure-driven water washing was conducted to replace the rinsing procedure using the same equipment with dynamic deposition. The pressure-driven washing was performed before crosslinking to ensure the soft state of the polyelectrolyte, and thus could form paths for water transport. The 1.5-bilayer membranes were prepared with static deposition and pressure-driven washing (Static-PW), dynamic deposition and pressure-driven washing (Dynamic-PW), static deposition and rinsing (Static-R), and dynamic deposition and rinsing (Dynamic-R). The separation performances of these membranes are presented in Table 1. A pressure of 0.2 MPa was used for all the pressure-driven procedures during the fabrication of all membranes. Among these membranes, the Static-PW membrane exhibited the highest water flux, while the Static-R membrane showed the lowest flux. At the same time, the rejection of the membranes was also improved by the pressure-driven washing procedure. These results further verify that the water-path could be constructed by pressure and lead to significantly increased flux, while for the dynamic deposited membranes, pressure-driven washing did not show obvious improvement in membrane performance. Water-paths were already formed during the dynamic deposition and further washing under pressure may compress the active layer and result in a denser structure, resulting in a slightly decreased flux and an increased rejection for membranes with pressure-driven washing. These results demonstrated that the dynamic deposition method improved the membrane fabrication efficiency by increasing the deposited polymer amount and also the density of the multilayer, compared to the static process. The optimized pressure could also lead to enhanced water flux by forming water paths in the membrane.

### 3.4. Retention of Different Salts

**Figure 8:**
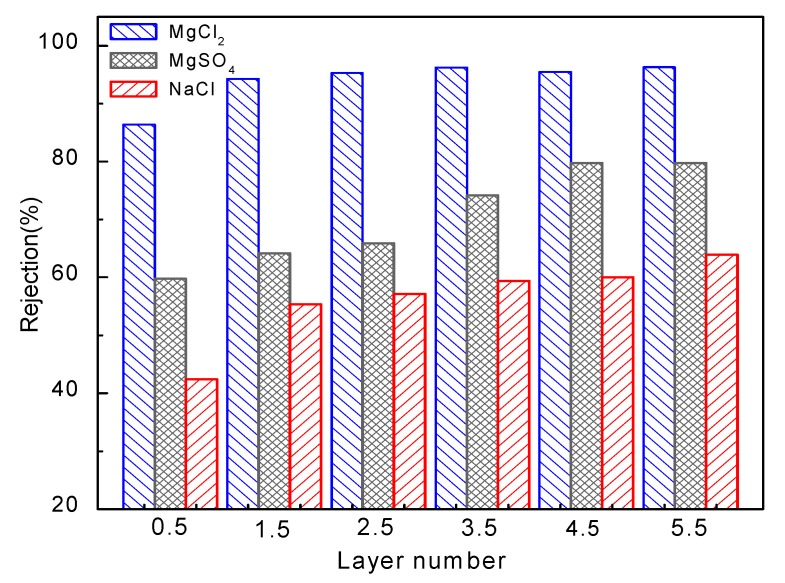
The retention of different salts of 0.2 MPa deposited membranes cross-linked with GA.

The retention of the GA cross-linked dynamic assembly membranes was investigated in terms of three different salts in 0.01 M: NaCl, MgCl_2_ and MgSO_4_. As shown in Figure 8, the membranes show the highest rejection of MgCl_2_, while exhibit the lowest rejection of NaCl. The Donnan and steric hindrance effects can interpret these. The cross-linked membranes exhibit a dense active layer and show higher retention to the larger Mg^2+^ than Na^+^. Furthermore, the surface of the PEI terminated membranes was positively charged at a pH close to neutral and exhibited decreased rejection of the monovalent Na^+^ than Mg^2+^ with higher valence. The higher rejection of MgCl_2_ compared with MgSO_4_ is attributed to the specific interaction between the bivalent sulfate ions and the positively charged membrane surface, which reduces the rejection of the magnesium by electrostatic screening [38]. It could also be seen in Figure 8 that the rejection of all salts increases with the bilayer number. The retention of the cross-linked membrane showed a rapid increase when the bilayer number increased from 0.5 to 1.5. For membranes with more bilayers, the rejection of MgCl_2_ and NaCl is relatively stable, while the rejection of MgSO_4_ increases with the layer of polyelectrolyte deposited (Figure 8). This again verified the electrostatic screening of the SO_4_^2−^ ions, so the steric hindrance effect caused by polyelectrolyte deposition and crosslinking plays a vital role in the retention of MgSO_4_.

## 4. Conclusions

A uniform composite NF membrane of PEI/PAA was prepared by dynamic deposition LBL self-assembly process on the surface of a hydrolyzed PAN substrate followed by chemical cross-linking. The membrane prepared with dynamic deposition exhibited a lower contact angle compared to those prepared by static adsorption. The cross-linked dynamically deposited multilayer membranes with a bilayer number of 1.5 exhibited an MgCl_2_ rejection of about 95%. Meanwhile, the non-cross-linked multilayer membranes rejected only just less than 70% of MgCl_2_ even with 5.5-bilayers. Therefore, crosslinking is a vital process to decreasing the required bilayer number of depositions. However, the cross-linked membranes exhibited an obviously declined flux. Crosslinking agent screening and crosslinking conditions optimization would be conducted in our further studies. The improvement of flux and the rejection of membranes with pressure-driven deposition under 0.2 MPa could be explained by a proposed water-path-forming mechanism. The positively-charged 1.5-bilayer NF membranes showed a higher rejection of MgCl_2_ than MgSO_4_ due to the electrostatic screening effect, while these membranes exhibited lowest rejection of NaCl because of the Donnan effect and size exclusion.

## Figures and Tables

**Table 1:** The separation performance of a membrane prepared with a different method.

	Static-PW	Static-R	Dynamic-PW	Dynamic-R
Flux (L/(m^2^·h·MPa))	31.2	16.8	20.9	22.7
Rejection (%)	83.2	74.0	96.5	95.3
